# Dynamic Effects of Self-Relevance and Task on the Neural Processing of Emotional Words in Context

**DOI:** 10.3389/fpsyg.2015.02003

**Published:** 2016-01-13

**Authors:** Eric C. Fields, Gina R. Kuperberg

**Affiliations:** ^1^Department of Psychology, Tufts University, MedfordMA, USA; ^2^Athinoula A. Martinos Center for Biomedical Imaging and Department of Psychiatry, Massachusetts General Hospital, CharlestownMA, USA

**Keywords:** emotion, ERP, language, late positive potential (LPP), late positive component (LPC), self-relevance, perspective, task

## Abstract

We used event-related potentials (ERPs) to examine the interactions between task, emotion, and contextual self-relevance on processing words in social vignettes. Participants read scenarios that were in either third person (other-relevant) or second person (self-relevant) and we recorded ERPs to a neutral, pleasant, or unpleasant critical word. In a previously reported study (Fields and Kuperberg, 2012) with these stimuli, participants were tasked with producing a third sentence continuing the scenario. We observed a larger LPC to emotional words than neutral words in both the self-relevant and other-relevant scenarios, but this effect was smaller in the self-relevant scenarios because the LPC was larger on the neutral words (i.e., a larger LPC to self-relevant than other-relevant neutral words). In the present work, participants simply answered comprehension questions that did not refer to the emotional aspects of the scenario. Here we observed quite a different pattern of interaction between self-relevance and emotion: the LPC was larger to emotional vs. neutral words in the self-relevant scenarios only, and there was no effect of self-relevance on neutral words. Taken together, these findings suggest that the LPC reflects a dynamic interaction between specific task demands, the emotional properties of a stimulus, and contextual self-relevance. We conclude by discussing implications and future directions for a functional theory of the emotional LPC.

## Introduction

Emotions have been described as “relevance detectors” (Frijda, 1986): if something in the environment is detected as being emotionally valenced or arousing, this indicates that it requires attention and further evaluation. Intuitively, however, what seems salient and worthy of further evaluation is influenced not only by the inherent properties of a particular emotional stimulus, but also by its perceived relevance to the comprehender given her current situation and goals. For example, when presented in isolation, the word “gun” may have negative connotations for some people, but positive connotations for others.^1^ However, almost anyone will find a gun pointed at him- or herself to be a negative experience, and this will likely be evaluated differently from seeing a gun pointed at a stranger. Further, our context and goals matter: a soldier will respond differently to the sight of someone with a gun than an unarmed civilian. In the present work, we used event-related potentials (ERPs), a direct online measure of neural activity, to ask how the interaction between goals (manipulated by task demands), emotion, and self-relevance influences our allocation of neural processing resources to emotional words within simple social vignettes that referred either to the comprehender or to another protagonist.

Our focus was on an ERP component known as the late positive component (LPC). The LPC has a parietal scalp distribution, begins at around 400–500 ms from stimulus onset, and extends for several hundred milliseconds (for a review, see Hajcak et al., 2012).^2^ It is generally larger to emotional than neutral stimuli and it is seen to both pictures (Olofsson et al., 2008; Hajcak et al., 2010) and words (Kissler et al., 2006; Citron, 2012). Its amplitude is enhanced by tasks that draw attention to emotional features of stimuli (e.g., Naumann et al., 1997; Fischler and Bradley, 2006; Schupp et al., 2007; Holt et al., 2009), and, more recently, it has also become clear that task demands can also influence the sensitivity of the LPC to different dimensions of emotional stimuli (Delaney-Busch et al., in press; see also Fischler and Bradley, 2006; Bayer et al., 2012). Importantly, the LPC evoked by emotional stimuli is not only influenced by their intrinsic emotional properties, but also by the context in which they are encountered. For example, the LPC evoked by emotional words can be influenced by the local sentence or discourse contexts in which they appear (e.g., Bartholow et al., 2001; Wang et al., 2013), as well as the broader contextual environment (e.g., Crites et al., 1995; Fogel et al., 2012). While the precise neurocognitive functions indexed by the emotion-sensitive LPC are still somewhat unclear, it is thought to reflect the capture and allocation of attentional resources by motivationally significant stimuli, leading to prolonged neural processing.

In a recent study, we examined the impact of contextual *self-relevance* on the LPC evoked by emotional and neutral words as participants read short social vignettes, with the goal of producing verbal continuations for each scenario (Fields and Kuperberg, 2012). The scenarios included neutral, pleasant, or unpleasant words, which potentially changed the meaning of the entire vignette, for example: “A man knocks on Sandra’s hotel room door. She sees that he has a gift/tray/gun in his hand.” In half of the scenarios the situations were made self-relevant by changing the context to the second person (see Brunyé et al., 2009), for example: “A man knocks on your hotel room door. You see that he has a gift/tray/gun in his hand.”

As expected, ERPs recorded on the critical words (CW; underlined above) showed a main effect of emotion with the pleasant and unpleasant words evoking a larger LPC than the neutral words. However, the interaction between emotion and self-relevance showed an unexpected but interesting result: the amplitude of the LPC evoked by neutral words was larger in the self-relevant scenarios than in the other-relevant scenarios. Self-relevance, however, had no effect on pleasant or unpleasant words.

We noted that many of our neutral scenarios could be interpreted as ambiguous in valence. Consider, for example, the scenario, “After dinner, you are involved in a discussion. Many of your remarks surprise people.” Here, it may not be immediately obvious why your remarks surprised people: was it because your comments were unexpectedly good, bad, or just unusual?^3^ Consequently, we argued that the selective effect of self-relevance on the neutral words may have been driven by participants’ continued attempts to assess their emotional valence (for effects of emotional ambiguity in ERPs, including the LPC, see Hirsh and Inzlicht, 2008; Gu et al., 2010; Tritt et al., 2012). In the self-relevant scenarios, participants likely invested additional processing resources to resolve this inherent valence ambiguity because of the additional demands of constructing a continuation consistent with their self-concept (Swann, 2011),^4^ whereas they had little motivation to go beyond the first interpretation that came to mind in the other-relevant scenarios. This difference in the demands imposed by the task in response to any valence ambiguity in the neutral scenarios was reflected by increased processing in the LPC time window.

If it is indeed the case that the particular pattern of LPC modulation observed in our previous study was driven by the interaction between emotion, self-relevance, and specific task goals, then we should see a different pattern of findings with different task demands. The aim of the current study was to determine if this was the case. To this end, a different set of participants viewed the same stimuli as we used in our previous study, but with different task requirements. Instead of producing a verbal continuation for each scenario, they read each scenario for comprehension and answered intermittent questions which encouraged deep discourse comprehension, but which did not refer specifically to the valenced aspects of the scenarios (see Holt et al., 2009; Delaney-Busch and Kuperberg, 2013; Paczynski et al., 2014; Fields and Kuperberg, 2015; Xiang and Kuperberg, 2015).

We hypothesized that, without the need to produce a specific continuation, there would be less demand on participants to interpret or disambiguate valence, and that we would therefore see a different allocation of neural processing resources, as reflected by the LPC. Specifically, we predicted that attention and processing resources would simply be directed to the most inherently motivationally relevant stimuli—in this case, the self-relevant *emotional* words. We therefore hypothesized that self-relevance would amplify the classic effect of emotion on the LPC (leading to larger differences between emotional and neutral words). Such a finding would be in line with previous studies examining the effects of self-relevance in two-word noun phrases with no overt task, which reported effects of emotion in the self-relevant condition, but not the other-relevant conditions (Herbert et al., 2011a,b), as well as with other studies reporting similar interactions between self-relevance and emotion (Li and Han, 2010; Shestyuk and Deldin, 2010; Schindler et al., 2014; see Discussion later in this manuscript).

Of course, as noted above, the idea that the LPC is influenced by task demands is not new. Indeed, some have suggested that it is related to the well-known P300 ERP component (see Discussion), which is evoked by perceptual oddball stimuli, particularly when they are task-relevant. Our aim here, however, was to understand whether and how task influences prolonged neural processing of emotional words in self-relevant contexts. Addressing this question is important because in real-world contexts the self-relevance and emotional impact of stimuli (and their interaction) will vary depending on the goals we have in a particular situation.

In order to directly compare the pattern of findings on the LPC using this comprehension task with those seen in our previous study using a production task (Fields and Kuperberg, 2012), we combined both datasets in a model in which task was analyzed as a between-subjects factor. To allow for a full and complete comparison between the studies, we report information from both studies in the Sections “Materials and Methods” and “Results” that follow. However, all methods and results for the production study are the same as those reported in Fields and Kuperberg (2012) and are reported in greater detail there.

## Materials and Methods

### Participants

Participants were recruited from postings on a university community website (tuftslife.com). As reported in Fields and Kuperberg (2012), 29 people originally participated in the production task experiment; three participants were excluded from analysis due to excessive artifact in the EEG, leaving 26 participants in the final analysis (15 females) between the ages of 18 and 29 (*M* = 20.7, *SD* = 2.30). Twenty-eight people originally participated in the comprehension task experiment; four participants were excluded from analysis due to excessive artifact in the EEG, leaving 24 participants (17 females) between the ages of 18 and 23 (*M* = 19.3, *SD* = 1.6).^5^ No individual participated in both experiments. All participants were right-handed native English speakers (having learned no other language before age 5) with no history of psychiatric or neurological disorders. Participants were paid for their participation and provided informed consent in accordance with the procedures of the Institutional Review Board of Tufts University.

### Stimuli

Stimuli are described in greater detail in Fields and Kuperberg (2012). Briefly, 222 sets of two-sentence scenarios were developed with Emotion (pleasant, neutral, and unpleasant) and Self-Relevance (self and other) conditions crossed in a 3 × 2 factorial design. The first sentence introduced a situation involving one or more people, only one of whom was specifically named (the protagonist). The situation was always neutral or ambiguous in valence. The second sentence continued the scenario and was the same across all emotion conditions except for one word, the CW, which was pleasant, neutral, or unpleasant. The part of speech of the CW was the same across the three Emotion conditions for each scenario: 37 of the scenarios had noun CWs, 54 had verb CWs, and 131 had adjective CWs. The named protagonist was male half the time and female the other half of the time. To create the self-relevant conditions, this named person was changed to “you” (previous work has shown that grammatical person is an effective manipulation of self-relevance: Brunyé et al., 2009). See **Table 1** for examples. The same set of stimuli was used in both experiments.

**Table 1:** Examples of two-sentence scenarios in each of the six conditions.

Other			Self		
Pleasant	Neutral	Unpleasant	Pleasant	Neutral	Unpleasant
A man knocks on Sandra’s hotel room door. She sees that he has a gift in his hand.	A man knocks on Sandra’s hotel room door. She sees that he has a tray in his hand.	A man knocks on Sandra’s hotel room door. She sees that he has a gun in his hand.	A man knocks on your hotel room door. You see that he has a gift in his hand.	A man knocks on your hotel room door. You see that he has a tray in his hand.	A man knocks on your hotel room door. You see that he has a gun in his hand.
Fletcher writes a poem for a class. His classmates think it is a very beautiful composition.	Fletcher writes a poem for a class. His classmates think it is a very intricate composition.	Fletcher writes a poem for a class. His classmates think it is a very boring composition.	You write a poem for a class. Your classmates think it is a very beautiful composition.	You write a poem for a class. Your classmates think it is a very intricate composition.	You write a poem for a class. Your classmates think it is a very boring composition.
Vince spends time with his relatives over the vacation. This turns out to be a wonderful experience for him in many ways.	Vince spends time with his relatives over the vacation. This turns out to be a characteristic experience for him in many ways.	Vince spends time with his relatives over the vacation. This turns out to be a disastrous experience for him in many ways.	You spend time with your relatives over the vacation. This turns out to be a wonderful experience for you in many ways.	You spend time with your relatives over the vacation. This turns out to be a characteristic experience for you in many ways.	You spend time with your relatives over the vacation. This turns out to be a disastrous experience for you in many ways.
After dinner, Lydia is involved in a discussion. Many of her remarks impress people.	After dinner, Lydia is involved in a discussion. Many of her remarks surprise people.	After dinner, Lydia is involved in a discussion. Many of her remarks hurt people.	After dinner, you are involved in a discussion. Many of your remarks impress people.	After dinner, you are involved in a discussion. Many of your remarks surprise people.	After dinner, you are involved in a discussion. Many of your remarks hurt people.
Carmelo has been in his current job for over a year. He learns that he is getting a bonus this month.	Carmelo has been in his current job for over a year. He learns that he is getting a transfer this month.	Carmelo has been in his current job for over a year. He learns that he is getting a pay-cut this month.	You have been in your current job for over a year. You learn that you are getting a bonus this month.	You have been in your current job for over a year. You learn that you are getting a transfer this month.	You have been in your current job for over a year. You learn that you are getting a pay-cut this month.

*The critical word is underlined (but did not appear underlined in the actual stimulus lists).*

#### Critical Word and Scenario Norms and Ratings

A series of norming studies of the stimuli were carried out via the internet. Inclusion criteria for participants in these ratings studies were the same as for the ERP experiments (see above). Means and standard deviations of all norms and ratings can be found in **Table 2**. Statistical analyses for CW length, CW concreteness, cloze probability, and constraint can be found in Fields and Kuperberg (2012). Briefly, stimuli were matched across conditions on all these features, except for concreteness where neutral words were slightly more concrete than pleasant and unpleasant words (this did not account for the unique effects of self-relevance on neutral words under the production task, see Fields and Kuperberg, 2012).

**Table 2:** Stimuli ratings and characteristics.

	Other pleasant	Other neutral	Other unpleasant	Self pleasant	Self neutral	Self unpleasant
Cloze Probability	3% (9%)	3% (7%)	3% (9%)	3% (8%)	3% (8%)	3% (7%)
Constraint	22% (13%)	22% (13%)	22% (13%)	22% (12%)	22% (12%)	22% (12%)
(log) HAL Frequency^∗^	8.39 (2.04)	8.47 (1.89)	8.28 (1.72)	–	–	–
Number of letters	7.67 (2.38)	7.48 (2.20)	7.14 (2.47)	–	–	–
Concreteness	3.45 (0.85)	3.72 (0.92)	3.54 (0.84)	–	–	–
Valence (word)	5.69 (0.55)	4.32 (0.56)	2.34 (0.57)	–	–	–
Arousal (word)	4.48 (0.80)	3.38 (0.64)	3.80 (0.63)	–	–	–
Valence (scenario)	5.25 (0.48)	4.12 (0.51)	2.37 (0.48)	5.40 (0.52)	4.17 (0.55)	2.24 (0.53)
Arousal (scenario)	3.61 (0.77)	3.22 (0.66)	3.84 (0.74)	3.87 (0.79)	3.49 (0.75)	4.11 (0.75)

*Means are shown with standard deviations in parentheses. Cloze probability and constraint are represented as the percentage of total responses from 29 subjects. Concreteness, valence, and arousal were all rated on seven point scales from least concrete (most abstract), very unpleasant, and least arousing, to most concrete, very pleasant and most arousing, respectively. “–” indicates that, for ratings conducted on the words in isolation from the scenario contexts, the values were the same in the self conditions as in the other conditions since the identical CWs were used (except for in six scenarios in which the verb was conjugated differently). ^∗^Some words did not exist in the HAL database and these were represented as null values in our calculations.*

Valence and arousal ratings were gathered for both the CWs in isolation and the scenarios (cut off after the CW). Valence ratings were as expected for both CWs and scenarios: the pleasant condition was rated as more pleasant than the neutral condition, which was rated as more pleasant than the unpleasant condition [*F*s > 1000, *p*s < 0.001]. In the scenarios, self-relevance amplified these differences, making pleasant scenarios more positive and unpleasant scenarios more unpleasant [Emotion × Self-Relevance interaction: *F*(2,442) = 26.50, *p* < 0.001].

As expected, there was a main effect of Emotion for the both the CW and scenario arousal ratings [*F*s > 70, *p*s < 0.001], with pleasant and unpleasant stimuli being rated as more arousing than neutral stimuli. The comparison of pleasant and unpleasant stimuli differed between the CW and scenario ratings: pleasant CWs were rated as more arousing than unpleasant CWs, but unpleasant scenarios were rated as more arousing than pleasant scenarios. There was no Emotion by Self-Relevance interaction in the scenario ratings [*F*(2,442) = 0.02, *p* = 0.980], but there was a main effect of Self-Relevance [*F*(1,221) = 162.71, *p* < 0.001] due to self-relevant scenarios being rated as more arousing than other-relevant scenarios.

### Procedure

#### Stimulus Presentation

In both experiments, scenarios were counterbalanced such that each scenario appeared in a different condition in each of six lists (thus appearing in all conditions across lists), and participants were randomly assigned to a list. Trials were randomly ordered within each list and the same lists with the same trial orderings were used in both experiments. All trials began with the word “READY” until the participant pressed a button to begin the trial. The first sentence then appeared in full until the participant pressed a button to advance. The second sentence began with a fixation cross displayed for 500 ms, followed by an interstimulus interval (ISI) of 100 ms, followed by each word of the sentence presented individually for 400 ms with an ISI of 100 ms. The final word of the scenario appeared on the screen for a longer duration of 750 ms, 400 ms ISI.

#### Task

In the first experiment, as described in Fields and Kuperberg (2012), a production task was used. Participants were instructed to verbally produce a single short sentence that followed naturally from the sentences they had just read (i.e., that continued the story). Participants were instructed to continue second-person (self-relevant) scenarios as if they were about themselves (i.e., in the first person). After the final word of each scenario, a question mark appeared on the screen, cuing participants to produce their verbal responses. Participants spoke into a microphone so that the experimenter was able to listen to their responses to ensure that they were in keeping with the content of each scenario. In addition, after 11 scenarios (randomly interspersed among each list), a yes or no comprehension question (as described below) followed the participant’s response, providing another objective measure of comprehension.

In the second experiment, the production task was eliminated and participants simply answered intermittent yes/no comprehension questions that appeared after forty of the scenarios (randomly interspersed). The question stayed on the screen until the participant gave an answer via button press. The question and its correct answer were the same across all conditions except where the self-relevance manipulation required changes. None of the questions referred to the valenced aspects of the scenarios. For example, the scenario “Casper is/You are new on campus. Everyone thinks he is/you are quite idiosyncratic/clever/dumb compared to most people.” was followed by the question “Did Casper/you go to this school last year?” with the correct answer being “no”.

#### ERP Acquisition and Processing

**FIGURE 1:**
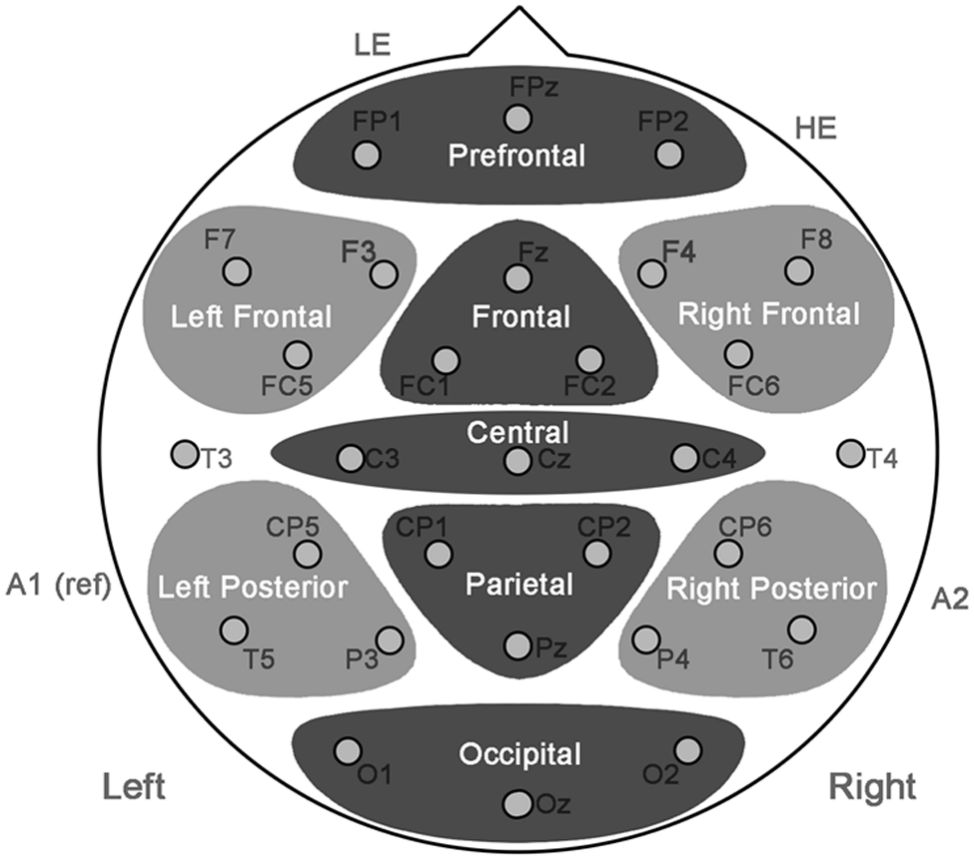
**Electrode montage with regions used for analysis.** For the purposes of statistical analyses, the scalp was divided into three-electrode regions. Regions in dark gray were part of the mid-regions omnibus ANOVA and regions in light gray were part of the peripheral regions omnibus ANOVA.

All equipment, acquisition parameters, and processing steps were the same between the two experiments. The EEG response was recorded from 29 tin electrodes in an elastic cap (Electro-Cap International, Inc., Eaton, OH; see **Figure 1**) referenced to the left mastoid. Additional electrodes were placed below the left eye and at the canthus of the right eye to monitor vertical and horizontal eye movements. The impedance was kept below 2.5 kΩ for mastoid electrodes, 10 kΩ for EOG electrodes, and 5 kΩ for all other electrodes. The EEG signal was amplified by an Isolated Biometric Amplifier (SA Instrumentation Co., San Diego, CA, USA), band pass filtered online at 0.01–40 Hz, and continuously sampled at 200 Hz.

The EEG was collected and processed using in-house software (available at: http://neurocoglaboratory.org/ERPSystem.htm). Segments from 100 ms before onset to 1100 ms after onset of each event were obtained. Trials with muscular and ocular artifact were identified and discarded using three algorithms: the first returns the number of time points within a given amplitude range of the minimum or maximum point of an epoch and is used to monitor for amplifier blocking or signal loss (i.e., a flat line); the second returns the difference between the maximum and minimum point of an epoch at the two EOG channels (independently) to monitor for horizontal and vertical eye movement; the third returns the difference of the mean difference and maximum difference between the electrode under the left eye and the electrode on the forehead above this eye and is used to identify blinks (which are characterized by opposite polarity shifts in these two channels). Appropriate thresholds for each of these algorithms were determined for each subject via visual inspection of the raw data (but were the same across all trials within each subject). Overall, 7.7% and 7.5% of trials were rejected for artifact for the production and comprehension tasks, respectively. The rejection rate did not differ across the Self-Relevance, Emotion, or Task conditions and there were no interactions between these factors [*F*s < 2.5, *p*s > 0.09].

#### ERP Analysis

For analysis purposes, the two studies were combined and Task was treated as a between-subjects variable. Averaged ERPs, time-locked to the CWs, were formed from trials remaining after artifact rejection and low pass filtered with a half-amplitude cutoff at 15 Hz. In order to examine how the modulation of the LPC varied across the scalp, the scalp was subdivided into three-electrode regions along its anterior–posterior distribution, at both mid-line and peripheral sites. Two omnibus ANOVAs, one covering mid-regions (dark gray in **Figure 1**) and another covering peripheral regions (light gray in **Figure 1**), were conducted with Emotion, Self-Relevance, Region, and Hemisphere (peripheral regions only) as within-subjects factors and Task as a between-subjects factor. For all tests of significance the Greenhouse and Geisser (1959) estimation of 𝜀 was used to correct the degrees of freedom (the original degrees of freedom are reported in the text). A significance level of alpha = 0.05 was used for all a priori comparisons.

## Results

The LPC was quantified by calculating the mean amplitude from 500 to 800 ms relative to a 100 ms prestimulus baseline. Analyses of other time windows are available in Fields and Kuperberg (2012) for the production task and Fields and Kuperberg (2015) for the comprehension task (see Footnote 5).

Combining both datasets, the Emotion × Self-Relevance × Task interaction was significant in both the mid-regions omnibus [*F*(2,96) = 7.49, *p* = 0.001, η^2^ = 0.135] and the peripheral regions omnibus [*F*(2,96) = 5.90, *p* = 0.004, η^2^ = 0.109]. The Emotion × Self-Relevance × Task × Region interaction was marginally significant in the mid-regions omnibus [*F*(8,384) = 2.32, *p* = 0.063, η^2^ = 0.046] and not significant in the peripheral regions omnibus [*F*(2,96) = 0.72, *p* = 0.489, η^2^ = 0.015]. Neither of these effects was further modulated by hemisphere in the peripheral regions ANOVA [*F*s < 1.6, *p*s > 0.20].

Below we follow-up these interactions by examining the Emotion × Self-Relevance interaction and Emotion × Self-Relevance × Region interaction in each task group separately. Additional analyses (including all main effects and interactions both in the combined analysis and each task group separately) are available as Supplementary Materials.

### Production Task

**FIGURE 2:**
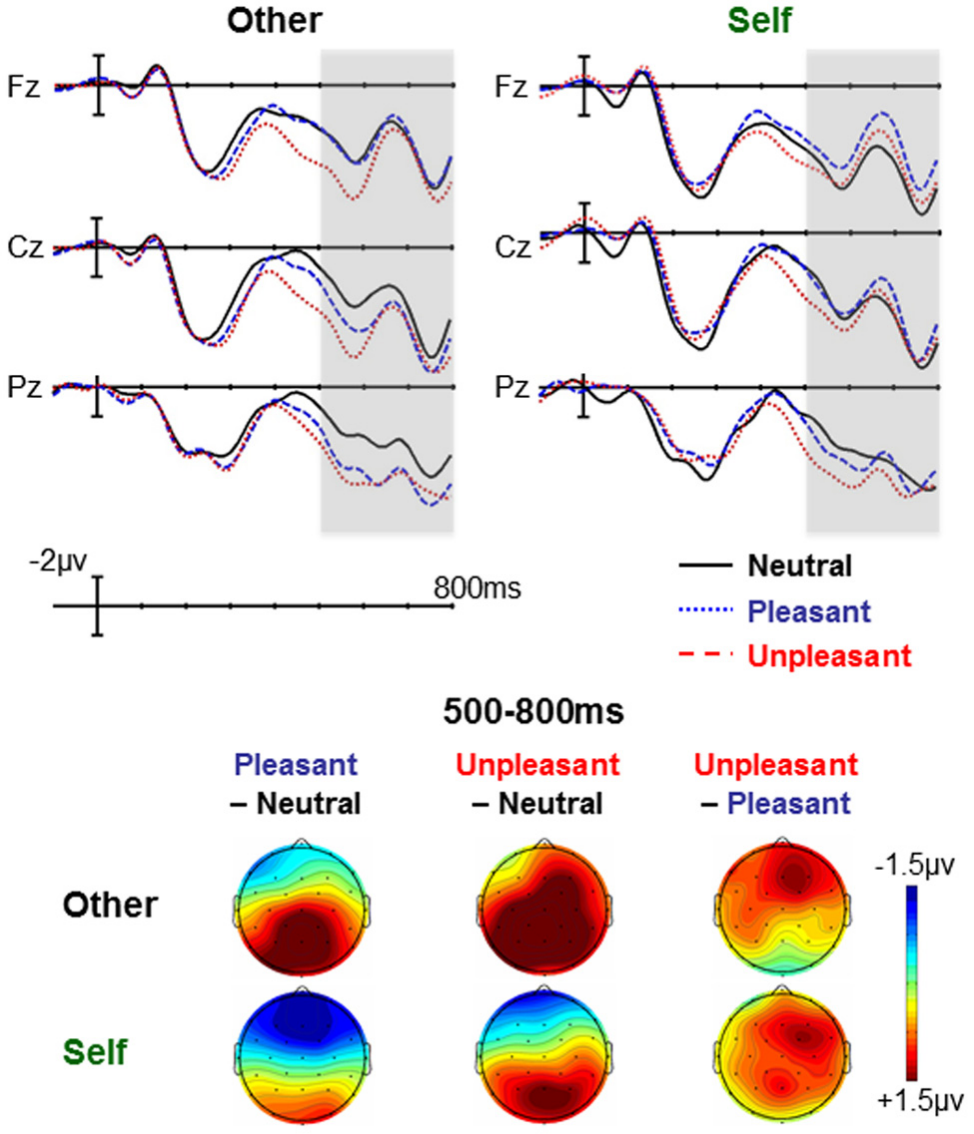
**Production task: Effects of emotion at each level of self-relevance.** Effects of emotion were seen in both other-relevant and self-relevant scenarios, but were smaller to self-relevant stimuli. This difference was driven by a larger positivity to neutral words in the self-relevant vs. other-relevant condition. The waveforms are low-passed filtered with a half-amplitude cut-off of 10 Hz for viewing purposes. Waveforms showing the effects of Self-Relevance at each level of Emotion are available in Fields and Kuperberg (2012).

As previously reported (Fields and Kuperberg, 2012), the Emotion × Self-Relevance interaction was significant in the mid-regions omnibus ANOVA [*F*(2,50) = 4.02, *p* = 0.026, η^2^ = 0.138] and marginally significant in the peripheral regions omnibus [*F*(2,50) = 2.70, *p* = 0.078, η^2^ = 0.098]. Both the mid-regions and peripheral regions omnibus ANOVAs showed significant effects of Emotion and/or significant Emotion × Region interactions in both the self-relevant and other-relevant scenarios, but these effects were larger in the other-relevant scenarios (see **Figure 2**).

This pattern, however, was driven entirely by the neutral words: self-relevant neutral words elicited a larger LPC than other-relevant neutral words (thus making them more similar to pleasant and unpleasant words) [mid-regions omnibus: *F*(1,25) = 20.18, *p* < 0.001, η^2^ = 0.447]. In contrast, pleasant and unpleasant words did not differ by self-relevance [*F*s < 0.4, *p*s > 0.55]. The effect of self-relevance for neutral words further interacted with Region [*F*(4,100) = 5.94, *p* = 0.008, η^2^ = 0.192] and follow-up ANOVAs in individual regions showed that the effect was strongest in the frontal region and was also significant in the prefrontal, central, and parietal regions. See Fields and Kuperberg (2012) for additional details.

### Comprehension Task

In the comprehension task study, the interaction between Emotion and Self-Relevance was significant in both the mid-regions ANOVA [*F*(2,46) = 3.73, *p* = 0.032, η^2^ = 0.140] and the peripheral regions ANOVA [*F*(2,46) = 3.32, *p* = 0.045, η^2^ = 0.126].

**FIGURE 3:**
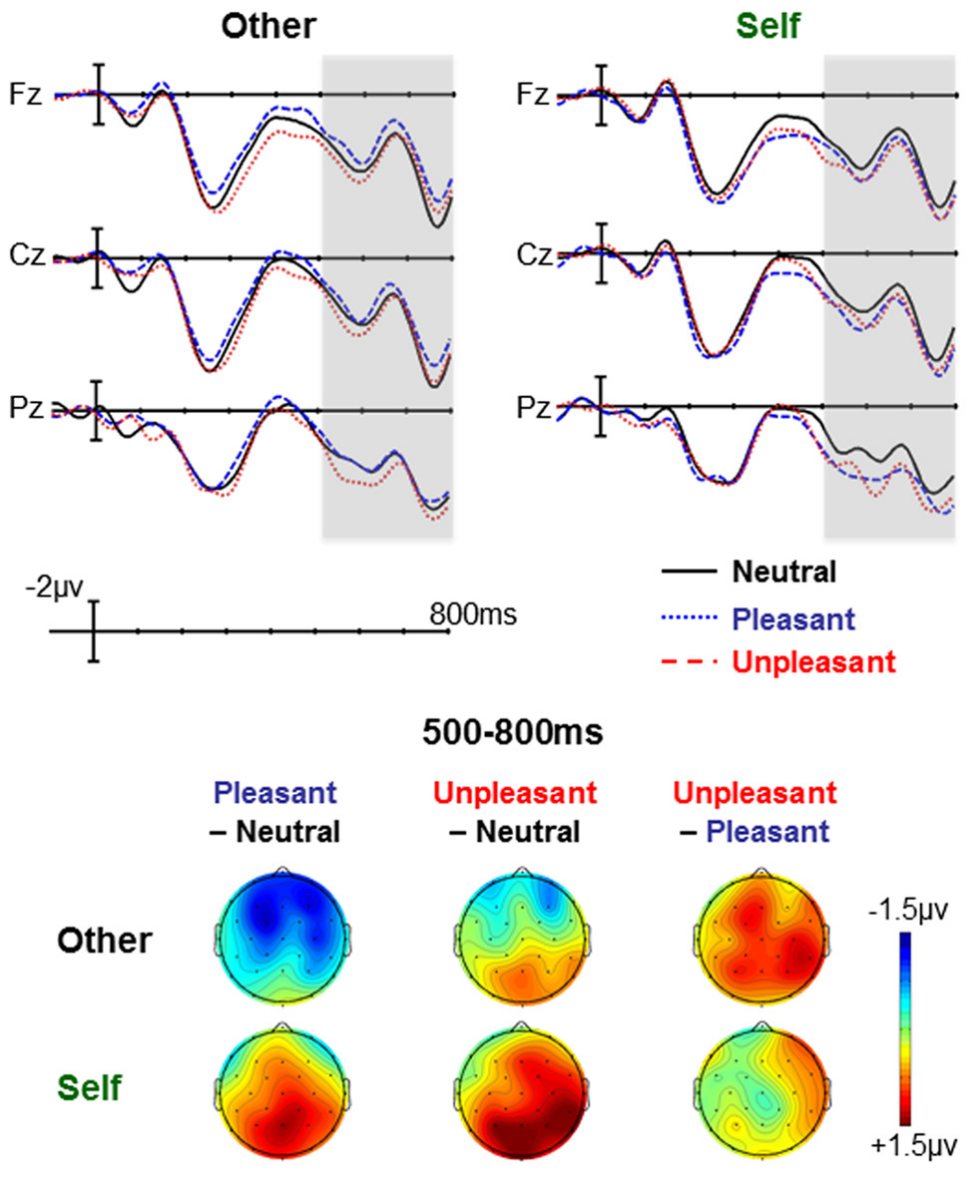
**Comprehension task: Effects of emotion at each level of self-relevance.** Effects of emotion were seen in self-relevant scenarios, but not other-relevant scenarios. The waveforms are low-passed filtered with a half-amplitude cut-off of 10 Hz for viewing purposes. Waveforms showing the effects of Self-Relevance at each level of Emotion are available in Fields and Kuperberg (2015).

However, the pattern of the effect was quite different from that observed with the production task: the interaction was primarily driven by a significant effect of Emotion in the self-relevant scenarios [mid-regions: *F*(2,46) = 3.86, *p* = 0.029, η^2^ = 0.144; peripheral regions: *F*(2,46) = 5.09, *p* = 0.011, η^2^ = 0.181], with no significant effect of Emotion in the other-relevant scenarios [mid-regions: *F*(2,46) = 2.26, *p* = 0.117, η^2^ = 0.089; peripheral regions: *F*(2,46) = 2.96, *p* = 0.064, η^2^ = 0.114], see **Figure 3**. Fisher–Hayter pairwise comparisons within the self-relevant scenarios confirmed that both pleasant and unpleasant CWs elicited a larger LPC than neutral CWs, but that the amplitude of the LPC to pleasant and unpleasant words did not differ.

This effect of Emotion (within self-relevant scenarios) had a centro-parietally centered, but broad, distribution (see **Figure 3**). It did not interact with the Region factor in the mid-regions ANOVA [*F*(8,184) = 2.05, *p* = 0.110, η^2^ = 0.082]. In the peripheral regions, the Emotion × Region interaction was significant [*F*(2,46) = 4.16, *p* = 0.023, η^2^ = 0.153] and follow-ups showed that the effect of Emotion was significant in the posterior region [*F*(2,46) = 7.60, *p* = 0.002, η^2^ = 0.248], but not the frontal region [*F*(2,46) = 1.77, *p* = 0.184, η^2^ = 0.071]. There were no significant interactions with the hemisphere factor [*F*s < 2.6, *p*s > 0.09].

When we broke down this interaction by examining the effect of Self-Relevance at each level of Emotion, the effect of Self-Relevance did not reach significance at any level of Emotion. There was a marginally significant effect of Self-Relevance on the pleasant words [*F*(1,23) = 3.47, *p* = 0.075, η^2^ = 0.131] and no effect on neutral or unpleasant words [*F*s < 1.4, *p*s > 0.25].

## Discussion

The aim of this work was to examine the influence of task demands and self-relevance on processing emotional words. We presented two-sentence social vignettes that were either contextually self-relevant or other-relevant and that contained a neutral, pleasant, or unpleasant CW in the second sentence. We compared our previous findings (Fields and Kuperberg, 2012) using a production task, with findings using a deep comprehension task (reported here). We observed an interaction between self-relevance and emotion in both studies, but the nature of this interaction was quite different depending on the task. With the production task, we observed a larger LPC to emotional words than neutral words in both the self-relevant and other-relevant scenarios, but this effect was smaller in the self-relevant scenarios because the LPC was relatively larger on neutral words (a larger LPC to self-relevant than non-self relevant neutral words). With the comprehension task, we only observed a larger LPC to emotional vs. neutral words in the self-relevant scenarios, and there was no effect of self-relevance on neutral words.

Previous work has shown that manipulations of both task (e.g., Naumann et al., 1997; Fischler and Bradley, 2006; Holt et al., 2009) and self-relevance (see Discussion below; Li and Han, 2010; Shestyuk and Deldin, 2010; Herbert et al., 2011a,b; Schindler et al., 2014) can enhance or attenuate the effect of emotion on the LPC. Other work has shown that which particular emotional features most strongly modulate the LPC can depend on which features a task draws attention to (Delaney-Busch et al., in press; see also Fischler and Bradley, 2006; Bayer et al., 2012). The present findings show something qualitatively different from either of these. The pattern of effects we observed went beyond simply enhancement or attenuation of the effects of emotion or even the relative effects of valence vs. arousal. Instead our studies show that that participants’ goals, the social relevance of the context, and the emotional properties of stimuli can interact in complex ways with regard to how and when neural resources are allocated in processing social situations as reflected by the LPC.

Below, we first offer an explanation of how and why the particular demands of the production and comprehension tasks led to the different patterns of LPC modulation we observed. We then discuss other possible differences between the two tasks that might have potentially influenced our findings. Finally, we turn to the more general implications of our findings for a functional interpretation of LPC, and briefly discuss some future directions of research.

### The Effect of Self-Relevance on the LPC Evoked by Emotional Vs. Neutral Stimuli

As described in the Section “Introduction” and in our previous work (Fields and Kuperberg, 2012), we suggest that the production task used in our previous study was critical in inducing the enhanced effect of self-relevance on neutral (but not emotional) words. This is because it encouraged participants to disambiguate the valence of the CW in order to produce a sensible and consistent continuation of the scenario as a whole. This was particularly important (or demanding) in the self-relevant condition because of participants’ desire to produce a continuation that was consistent with their self-concept (Swann, 2011). We argued that the larger LPC reflected this prolonged enhanced neural processing to self-relevant neutral.^6^

In the present study, participants were required to comprehend each sentence deeply (in order to respond to comprehension questions). However, these questions did not refer to the valenced aspects of the scenarios, and there was no additional requirement to produce a specific continuation for each scenario. Therefore, there was nothing to motivate participants to disambiguate the valence of the neutral words. In this situation, we suggest that processing resources were simply allocated to the stimuli that were most inherently motivationally relevant and attention grabbing. These were the self-relevant emotional CWs. Indeed, in the self-relevant scenarios, the LPC was larger to the emotional than the neutral words.

This effect of self-relevance enhancing the effect of emotion on the LPC in the comprehension task is consistent with previous behavioral work that reports greater changes in participants’ emotional states after they read self-relevant emotional texts vs. non-self-relevant emotional texts (Brunyé et al., 2011). Similarly, the results of our rating studies (see Materials and Methods) showed that self-relevance led to pleasant stimuli being rated as more positive and unpleasant stimuli being rated as more negative. This pattern is also consistent with some previous ERP studies that have examined the interaction between self-relevance and emotion. For example, Shestyuk and Deldin (2010) saw differences between pleasant and unpleasant words (they did not include neutral words) on the LPC when words were judged for self-relevance but not when they were judged for their relevance to Bill Clinton. In a related study, Schindler et al. (2014) showed participants (with no overt task) trait adjectives under either a) a condition where a second person was supposedly judging whether the adjective applied to the participant or b) a condition where a computer was simply randomly presenting the words. They only found effects of emotion for words in the judgment (i.e., self-relevant) condition. In work more similar to our own, Herbert and colleagues (Herbert et al., 2011a,b) report two studies in which participants passively read (with no additional task) emotional and neutral words preceded by first-person and third-person pronouns. They found effects of emotion on the LPC only for words preceded by the first-person pronouns (see also Li and Han, 2010). Thus, in all these studies, just as in the present study, effects of emotion were seen in the self-relevant condition, but not in the non-self-relevant conditions. We now turn to possible reasons for this.

### The Effect of Emotion on the LPC Evoked by Non-Self-Relevant Stimuli

With the production task, we saw a larger LPC to emotional than neutral words following both the self-relevant *and* other-relevant contexts. These effects are consistent with a large body of ERP studies that have reported emotion effects on the LPC in single words (reviewed in Kissler et al., 2006; Citron, 2012) and to emotional words in non-self-relevant contexts (e.g., Bartholow et al., 2001; Holt et al., 2009; Bayer et al., 2010; Delaney-Busch and Kuperberg, 2013). Thus, it is striking that in the studies described above (Li and Han, 2010; Shestyuk and Deldin, 2010; Herbert et al., 2011a,b; Schindler et al., 2014) and in the present study using the comprehension task, a larger LPC was observed to emotional (vs. neutral) words *only* in self-relevant contexts.

We argue that this apparent discrepancy can be explained within the broad dynamic framework we have been describing. Specifically, we suggest that allocation of attention and resources is not only a function of the inherent emotional salience of stimuli, overt task demands, and self-relevance of the immediate context, but it is *also* a function of the broader context of the environment (in this case, the surrounding stimuli within the experimental context). This influence of the broader environmental context can be understood at an intuitive level. What seems salient and important enough to garner attention in one situation may not be relevant in another: a spider discovered in your living room may dominate your attention under normal circumstances, but if your house is on fire, it’s not likely to receive much of your attention. This sensitivity to broader environmental context was recently illustrated in a study by Fogel et al. (2012), who showed that differences between emotional words and neutral words on the LPC disappeared when highly salient taboo words were mixed into the stimuli, presumably because the standard emotional words lost their ability to draw special attention in the presence of the more arousing taboo words (see also Crites et al., 1995).

We suggest that, with the comprehension task in the present work, as well as in previous studies (Li and Han, 2010; Shestyuk and Deldin, 2010; Herbert et al., 2011a,b; Schindler et al., 2014), the non-self-relevant scenarios, even when emotional, lost their ability to draw additional attention in the presence of self-relevant emotional scenarios. In contrast, when emotional properties were task-relevant, as we have argued they were for the production task, attention was allocated to the emotional properties of words across conditions, leading to an enhanced LPC for emotional words regardless of their self-relevance. This explanation, of course, remains somewhat speculative. Future work is needed to systematically explore the effects of task demands on the emotional LPC to non-self-relevant stimuli in the presence of self-relevant stimuli.

### Other Differences Between the Production and Comprehension Tasks

In the discussion above, we attributed the different patterns of ERP modulation across the two tasks to the fact that the production but not the comprehension task encouraged participants to disambiguate the valence of the self-relevant neutral words. We now consider other differences between these two tasks that might have contributed to the different pattern of effects seen in the two experiments.

One possibility is that the production task encouraged deeper semantic processing of the scenarios as a whole than the comprehension task. We think that this difference is unlikely to have driven the different pattern of ERP findings for two main reasons. First, in the comprehension task, participants answered intermittent comprehension questions that required them to deeply comprehend and build a situation model of each discourse scenario. These questions were written such that they required information from different parts of the scenario and often required an inference based on the situation model described by the scenario. This meant that participants could not simply rely on any superficial semantic strategy to correctly answer these questions (see also Holt et al., 2009; Delaney-Busch and Kuperberg, 2013; Paczynski et al., 2014; Fields and Kuperberg, 2015; Xiang and Kuperberg, 2015). Second, while depth of semantic processing can influences ERPs, particularly on the N400 (e.g., Chwilla et al., 1995), it is not clear why it would generate the specific effect we observed on neutral scenarios unless it was because they were harder to process, perhaps due to ambiguity, which is similar to the explanation that we provide.

A second difference between the production and comprehension tasks is that the former required participants to plan their production utterances, whereas this was not necessary with the comprehension task. It is possible that such planning overlapped with the processing reflected in the ERPs we recorded. But this simply raises the question of why such planning would require greater processing specifically in the self-relevant neutral scenarios. We have argued it did so because of the motivation to produce a continuation consistent with the participants’ self-concept in the presence of ambiguity.

Third, it is possible that, in reading the self-relevant scenarios, participants failed to adopt a self-relevant perspective in the comprehension task, as they did in the production task (since in the production task they had to produce a continuation specifically about themselves). Once again, however, this does not easily account for our findings. First, there is independent evidence that participants can and do automatically adopt self-relevant perspectives in reading second person scenarios during comprehension (Brunyé et al., 2009, 2011). In addition, it is difficult to explain other aspects of our results if participants did not interpret the second person scenarios as self-relevant with the comprehension task: what would account for the modulation of the effect of emotion by the self-relevance factor? One might argue that our findings were driven by the requirement to explicitly plan a self-relevant responses in the production task that was enhanced in neutral scenarios—an explanation that is again very similar to the one that we offer.

There are surely other important differences between the tasks as well. But any explanation of the present findings must explain how these differences interact with both emotion and self-relevance to produce the specific pattern of findings observed across our two studies. It will be important for future studies to find novel ways to test the explanations presented here.

### Implications and Open Questions

Taken together with the previous literature, our work suggests that the allocation of resources to emotional and self-relevant stimuli reflected by the LPC is highly dynamic. While previous work has suggested that the LPC may reflect or be modulated directly by the emotional properties of stimuli (e.g., Cuthbert et al., 2000; Olofsson et al., 2008), our results and others clearly show that there is no one-to-one relationship between any given property of an eliciting stimulus (valence, arousal, self-relevance, ambiguity, etc.) and LPC amplitude. In this work, we manipulated the potential influence of three factors: (1) the “inherent” emotional salience of the stimulus itself (a function of enduring biological and social motivations), (2) the local context in which a particular incoming stimulus is encountered (in this design, the self-relevance of the discourse social vignettes), and the 3) the situation-specific goals provided by a particular task. We also discussed the role of a fourth factor: the broader experimental context. As we have discussed, none of these factors is either sufficient or necessary to evoke an LPC effect; nor are their effects simply additive. Rather, they interactively influence LPC amplitude.

This, of course, raises the question of what functional neural mechanism the LPC actually reflects. That is, what neurocognitive process is being modulated by the factors discussed above and how do these factors interact to influence this process? We suggest that one clue into the nature of this mechanism comes from the striking resemblance between the factors known to affect the LPC and the factors that are known to modulate the widely studied P300 component.^7^ The P300 is a positive-going component that is famously evoked by stimuli that are surprising or unexpected in their experimental context. This effect is modulated by multiple factors including local sequence effects, global probability, contingencies between stimuli, experimental instructions, the perceived value of a stimulus, and task relevance (for reviews of factors affecting the P300, see Donchin and Coles, 1988; Johnson, 1988; Polich, 2012). Importantly, despite its name, the P300 peaks at a range of latencies. Indeed, in response to more complex manipulations, such as those based on the semantic content of words, it tends to peak in the LPC time window (Kutas et al., 1977; reviewed by Donchin and Coles, 1988; Polich, 2012). In other words, the P300 is morphologically quite similar to the LPC, in addition to being sensitive to some of the same manipulations. While the links between the P300 evoked by oddball stimuli and LPC produced by motivationally relevant stimuli have often been noted in the literature (e.g., Citron, 2012; Hajcak et al., 2012; Weinberg et al., 2012), the relationship between these components has not been a topic of direct investigation or in-depth theoretical discussion.

To the extent that the many similarities between the P300 and the emotional LPC go beyond a superficial resemblance, the theoretical literature on the P300 will provide insights into the function of the LPC. A number of functional theories of the P300 have been proposed (Donchin, 1981; Donchin and Coles, 1988; Nieuwenhuis et al., 2005; Polich, 2007; Twomey et al., 2015). Such theories have often related the P300 to a process of maintaining an accurate model of the current environment. The P300 is thought to be evoked to the extent that incoming information leads us to *update* this internal model (the representation of the broader environmental context; Donchin and Coles, 1988). The literature on Bayesian generative models of cognition may offer a more contemporary view of this “context updating” process (see Perfors et al., 2011; Qian et al., 2012; Clark, 2013; Friston et al., 2015). In this framework, task relevance modulates the P300 because our model of the environment is tailored to our goals and motivations—that is, we are trying to build a model of the environment that helps to achieve the goals of whatever task we are engaged in.

It is intuitive that emotional stimuli might also be associated with this sort of model updating process. In complex, noisy, and ever-changing environments, we constantly need to monitor what stimuli are relevant or not, and which actions will be most useful for pursuing our goals. As noted at the beginning of this paper, emotions can act as “relevance detectors” (Frijda, 1986), telling us which information is relevant to our goals and motivations. They therefore indicate which information is most important to integrate into our context model, or when we might need to adapt our current model or switch to a new model. And, as we have shown in the present study, what is relevant will depend on many interacting factors, and the LPC will therefore be sensitive to all these factors—not simply the valence or arousal of the eliciting stimulus.

Future work should further examine the relationship between the P300, LPC, and context updating related processes. One way to do this is to carefully examine how the LPC responds to factors known to modulate the P300, such as stimulus probability. Computational modeling may be helpful to understanding how various factors affecting these components are likely to interact and why. In addition, given the difficulty of identifying when components are the same vs. distinct (Kappenman and Luck, 2012), this work will also likely be aided by examining the LPC (and comparing it to the P300) using techniques with higher spatial resolution such as MEG and fMRI (e.g., Liu et al., 2012; Sabatinelli et al., 2007, 2013), as well as complementary ways to examine the EEG such as time-frequency analyses.

### Summary and Conclusion

In sum, we have shown a complex three-way interaction between the emotional properties of a stimulus, the self-relevance of its local context, and task demands when participants process socially relevant real-world vignettes. When participants were asked to produce sentences to continue each scenario, self-relevance enhanced the amplitude of the LPC specifically on neutral words. When participants simply answered questions that did not require attention to the self-relevant or emotional aspects of the scenarios, self-relevance enhanced the typical effect of emotion on the LPC. These results suggest that there is no one-to-one relationship between the emotional properties (or self-relevance) of an eliciting event and its effects on neurocognitive processing. They suggest that we allocate attention and processing to emotional stimuli in a highly dynamic fashion that is calibrated to the demands of a given situation, and they support the view that the LPC is triggered by a highly dynamic computational mechanism. One candidate for the function of this mechanism is adapting a current model or switching to a new internal model that best represents our contextual environment in relation to our goals, enabling us to do better job at predicting incoming information in the future, as proposed by theories of the P300.

## Conflict of Interest Statement

The authors declare that the research was conducted in the absence of any commercial or financial relationships that could be construed as a potential conflict of interest.

## Footnotes

1 It has been pointed out to us that the idea of having a generally positive view of guns may seem strange in some cultural contexts, but we can assure readers that in the U.S. gun ownership is simply a hobby for many people and, for better or worse, a generally positive view of guns is not uncommon.

2 Some research suggests there may be multiple, related emotion-sensitive late positivities (Delplanque et al., 2006; Foti et al., 2009; MacNamara et al., 2009; Hajcak et al., 2012; Matsuda and Nittono, 2015). In practice these are often difficult to distinguish, and indeed most widely-distributed later components of the ERP have multiple underlying neural sources (Luck, 2014). Here we use the term “late positive component” as a general term for emotion-sensitive positivities peaking after approximately 400 ms.

3 As another example: “You have been in your current job for a over a year. You learn that you are getting a bonus/transfer/pay-cut this month.” While a bonus or pay-cut are clearly good and bad respectively, a transfer is more ambiguous in this particular context. Did you want a transfer? Is the transfer in response to good or bad performance? On closer examination, it turned out that many of the neutral scenarios were ambiguous in similar ways. See **Table 1** and the Supplementary Materials to Fields and Kuperberg (2015) for more examples.

4 Independent valence ratings of these responses showed that responses to self-relevant scenarios were more positive than those to non-self-relevant scenarios, consistent with the widely observed self-positivity bias (Taylor and Brown, 1988; Alicke and Govorun, 2005; Fields and Kuperberg, 2015). Notably, this also held true specifically for neutral scenarios. These results support the assertion that participants tended to produce continuations that were consistent with their self-concept.

5 These participants, and the ERPs we report from them, are the same as those reported in another paper by Fields and Kuperberg (2015), which focused on how the N400 was modulated in response to the self-positivity bias. We decided to discuss the N400 and LPC findings in separate manuscripts because, as can be seen by comparing the present paper to Fields and Kuperberg (2015), we see the results on these two components as being relevant for two different literatures and sets of theoretical questions. However, it is important to note that the results on the N400 and the LPC displayed qualitatively different patterns that rule out component overlap explaining either effect (see discussion in Fields and Kuperberg, 2015).

6 It is worth noting that this effect had a more frontal distribution than the standard posterior effect of emotion on the LPC. This suggests that the nature of the further processing induced by these particular stimulus and task conditions may have been distinct from that usually seen to emotional stimuli that are not ambiguous. For discussion of this issue, see Fields and Kuperberg (2012).

7 Following common usage, we use the term P300 to refer to the component sometimes more specifically identified as the “P3b” (see Polich, 2012).
